# Prunetin Relaxed Isolated Rat Aortic Rings by Blocking Calcium Channels

**DOI:** 10.3390/molecules23092372

**Published:** 2018-09-17

**Authors:** Bumjung Kim, Cheolmin Jo, Ho-Young Choi, Kyungjin Lee

**Affiliations:** 1 Department of Herbology, College of Korean Medicine, Kyung Hee University, Seoul 02447, Korea; ori-pharm@hanmail.net (B.K.); hychoi@khu.ac.kr (H.-Y.C.); 2 Department of Herbology, Graduate School, Kyung Hee University, Seoul 02447, Korea; chocm456@naver.com

**Keywords:** prunetin, hypertension, blood pressure, vasorelaxation, calcium channels

## Abstract

Prunetin, a component of herbal medicines and various foods, such as pea, peach, cherry, and *Prunus yedoensis*, is a useful pharmacological compound. We previously reported the potent vasorelaxant effect of the bark of *P. yedoensis*. Therefore, we investigated the vasorelaxant activities of prunetin on isolated rat aortic rings and hypotensive activity on spontaneously hypertensive rats (SHR) in this study. In the present study, prunetin (1–30 μg/mL) relaxed isolated rat aortic rings pre-contracted by phenylephrine (PE) in a concentration-dependent manner. Pre-incubation with prunetin (3 and 10 μg/mL) inhibited vasoconstriction induced by the supply of Ca^2+^ in rat aortic rings pre-contracted with PE or KCl in a Ca^2+^-free Krebs–Henseleit (KH) buffer. Prunetin (10 μg/mL) pre-treatment also inhibited caffeine-induced contraction of aortic rings in a Ca^2+^-free KH buffer. To investigate the hypotensive effect of prunetin, the systolic blood pressure (SBP) of the SHR was measured by using a tail cuff assay. The SBP of SHR was significantly lower in the prunetin (25 mg/kg)-treated group. These results suggested that prunetin decreased blood pressure and relaxed blood vessels by blocking receptor-operated calcium channels, voltage-dependent calcium channels, and ryanodine receptor channels.

## 1. Introduction

According to the World Health Organization (WHO), in 2015, 1.13 billion people worldwide had high blood pressure (systolic blood pressure [SBP] ≥ 140 and/or diastolic blood pressure [DBP] ≥ 90) [1]. High blood pressure is one of the major global risk factors for mortality from conditions such as coronary heart disease, heart failure, peripheral vascular disease, renal impairment, retinal hemorrhage, visual impairment, ischemic, and hemorrhagic stroke. Blood pressure levels below 140/90 are associated with a decreased incidence of stroke and coronary heart disease [1]. Thus, the maintenance of healthy blood pressure is required to reduce cardiovascular complications.

It is important to manage lifestyle factors, such as weight, diet, exercise, alcohol consumption, and tobacco smoking, to prevent and control hypertension. If blood pressure cannot be controlled by these nonpharmacological treatments, hypertensive drugs can be used, such as calcium channel blockers, thiazide diuretics, angiotensin receptor blockers, angiotensin-converting enzyme inhibitors, and β-blockers. Currently, most patients with hypertension use these medications to control their blood pressure, but there are many foods and herbal medicines that can prevent or control hypertension; representative antihypertensive plants include green asparagus [2], celery [3], red wine [4], and safflower [5].

Prunetin, a typical phytoestrogen, is an *O*-methylated isoflavone that exhibits various pharmacological effects, such as anti-inflammatory activity [6,7], anti-obesity/anti-adipogenic activity [8], chondroprotective activity [9], osteoinductive effects [10], cytotoxic activity in human hepatoma HuH-7 cells [11], α-glucosidase inhibitory effect [12], and improvements of fitness and lifespan in male *Drosophila melanogaster* [13].

Prunetin is found in many plants used for medicines and foods, including pea [14], peach [15], oregon cherry [16], skimmed cheese, whole cheese, cow kefir, goat kefir [17], *Prunus avium* [10], *Andira surinamensis* [18], *Butea superba* [19], *Dalbergia sympathetica* [20], *Ficus nervosa* [21], *Pterospartum tridentatum* [22], and *Pycnanthus angolensis* [23]. 

In a previous study, we reported the potent vasorelaxant effect of the bark of *P. yedoensis* [24], of which prunetin is one of the major compounds. Therefore, we hypothesized that prunetin will also exert vasorelaxant and hypotensive activities, and therefore evaluated the vasorelaxant activities of prunetin on isolated rat aortic rings and the hypotensive activity in spontaneously hypertensive rats (SHR).

## 2. Results

### 2.1. Vasorelaxant Effects of Prunetin in Presence or Absence of Vascular Endothelium

**Figure 1:**
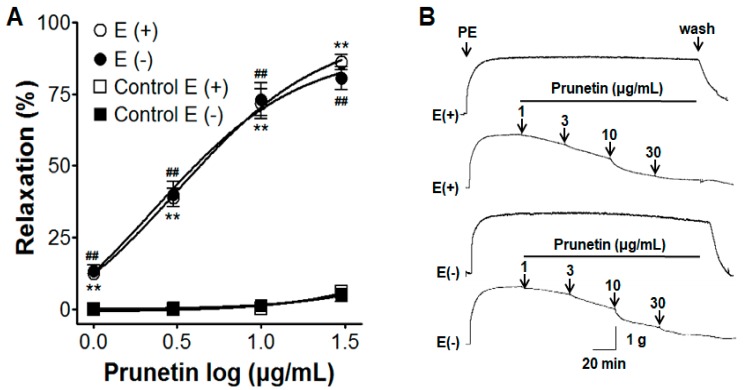
The concentration-response relaxation effect of prunetin on aortic rings pre-contracted by phenylephrine (PE, 1 μM) (**A**) with [(E+)] or without [(E−)] vascular endothelium; (**B**) Representative traces under the indicated conditions. Values are expressed as mean ± standard error of mean (SEM) (*n* = 4). ** *p* < 0.01, ^##^ *p* < 0.01 vs. control.

Regardless of the presence of the endothelium, prunetin caused a concentration-dependent relaxation response in aortic rings pre-contracted by phenylephrine (PE, 1 μM). The maximal relaxant effect of prunetin on PE-induced contraction was 86.2% ± 2.7% and 80.5% ± 4.1% for endothelium-intact and endothelium-denuded aortic rings, respectively (Figure 1).

### 2.2. Vasorelaxant Effects of Prunetin on Extracellular Ca^2+^-Induced Contraction

**Figure 2:**
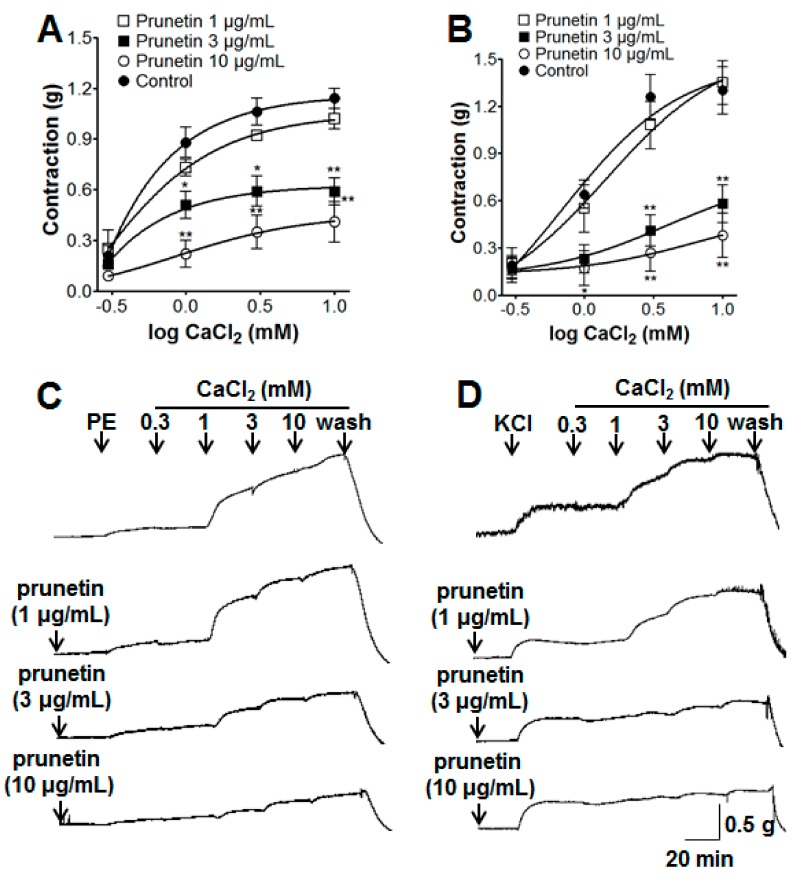
The inhibitory effect of prunetin (1–10 μg/mL) on the contraction induced by CaCl_2_ on endothelium-denuded aortic rings pre-contracted by phenylephrine (PE, 1 μM) (**A**,**C**) or KCl (60 mM) (**B**,**D**) in the presence or absence (control) of prunetin. (C) and (D) are representative traces under the indicated conditions. Values are expressed as mean ± SEM (*n* = 4–6). * *p* < 0.05, ** *p* < 0.01 vs. control.

A gradual increase in tension of endothelium-denuded aortic rings was induced by the addition of CaCl_2_ (0.3–10 mM) in Ca^2+^-free Krebs–Henseleit (KH) buffer. Under the conditions of endothelium denudation, prunetin (3 and 10 μg/mL) pre-treatment significantly attenuated the contraction induced by CaCl_2_ (10 mM), the contraction observed with prunetin (10 μg/mL) was 0.41 ± 0.12 g and 0.38 ± 0.14 g compared with the control group 1.14 ± 0.06 g and 1.30 ± 0.15 g on aortic rings pre-contracted by PE or potassium chloride (KCl), respectively (Figure 2).

### 2.3. Vasorelaxant Effects of Prunetin on Intracellular Ca^2+^ Release

**Figure 3:**
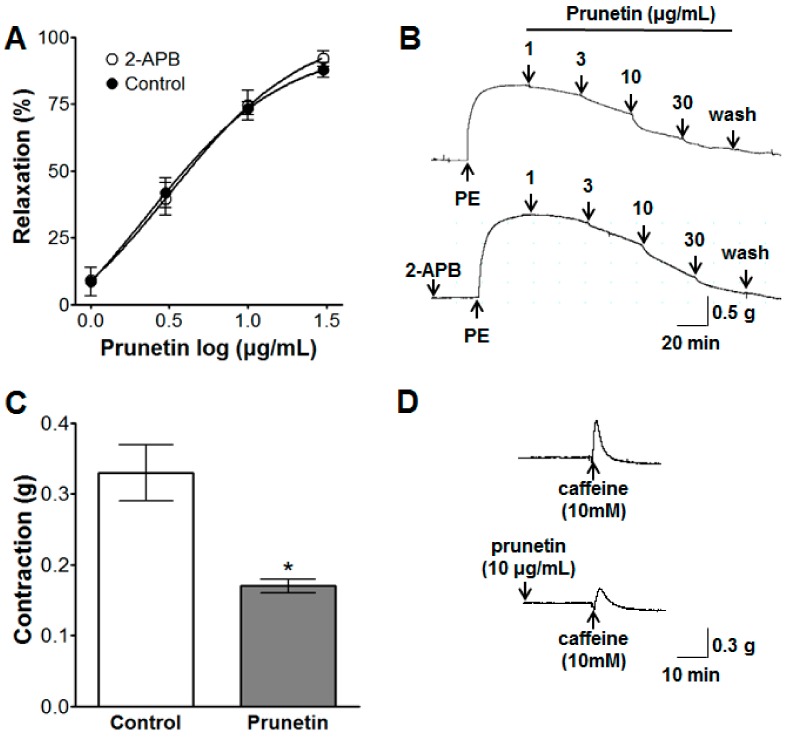
The vasorelaxant effect of prunetin (1–30 μg/mL) on vessels pre-contracted by phenylephrine (PE, 1 μM) with or without pre-treatment with 2-aminoethoxydiphenyl borinate (blocker of store-operated Ca^2+^ entry, 2-APB) (**A**,**B**) and the inhibitory effect of prunetin (10 μg/mL) on vasocontraction induced by caffeine (RyR agonist, 10 mM) (**C**,**D**). (**B**,**D**) are representative traces under the indicated conditions. Values are expressed as mean ± SEM (*n* = 4–5). * *p* < 0.05 vs. control.

Prunetin (1–30 μg/mL) altered the PE-induced (1 μM) contraction when the samples were pre-incubated with 2-aminoethoxydiphenyl borinate (2-APB, 10 μM) for 20 min. In addition, under conditions of endothelium denudation, pre-incubation with prunetin (10 μg/mL) inhibited caffeine-induced (10 mM) contractions (Figure 3).

### 2.4. Vasorelaxant Effects of Prunetin on the Rho-Kinase Pathway 

**Figure 4:**
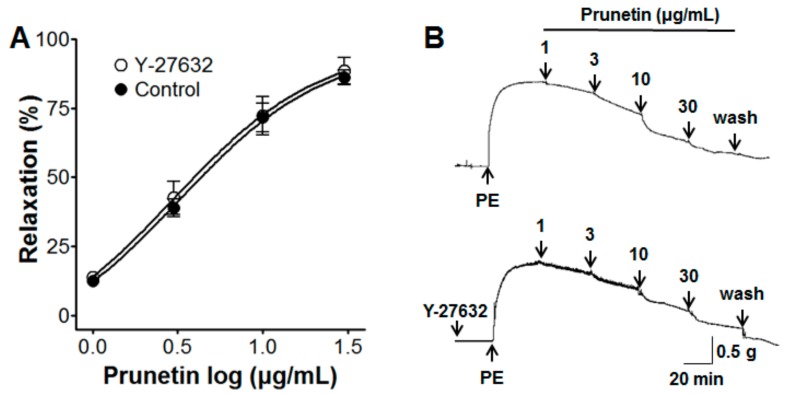
Concentration-response relaxation effect (**A**) and representative traces of the effect (**B**) of prunetin on endothelium-denuded aortic rings pre-contracted by phenylephrine (PE, 1 μM) in the presence or absence (control) of Y-27632 (Rho-kinase inhibitor, 1 μM). Values are expressed as mean ± SEM (*n* = 4).

Under the conditions of endothelium denudation, the vasorelaxant effects of prunetin (1–30 μg/mL) on PE-induced (1 μM) contraction were not changed by the addition of Y-27632 (1 μM) (Figure 4).

### 2.5. Vasorelaxant Effects of Prunetin on Aortic Rings with Vascular Endothelium Pre-Treated by Various K^+^ Channel Blockers

**Figure 5:**
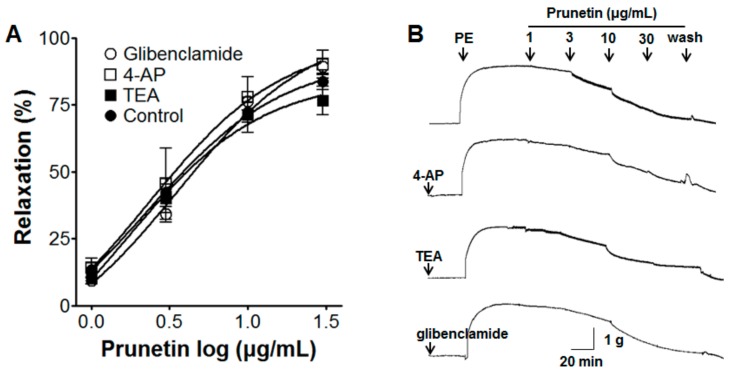
Concentration-response relaxation effect (**A**) and representative traces of the effect (**B**) of prunetin on endothelium-intact aortic rings pre-contracted by phenylephrine (PE, 1 μM) with or without (control) glibenclamide (K_ATP_ channel blocker, 10 μM), 4-aminopyridine (K_V_ channel blocker, 4-AP, 1 mM), or tetraethylammonium (BK_Ca_ channel blocker, TEA, 5 mM). Values are expressed as mean ± SEM (*n* = 4–6).

Incubation with a K^+^ channel blocker, such as glibenclamide (10 μM), 4-aminopyridine (4-AP, 1 mM), or tetraethylammonium (TEA, 5 mM), did not alter prunetin-induced relaxation of endothelium-intact aortic rings pre-contracted by PE (1 μM) (Figure 5).

### 2.6. Hypotensive Effects of Prunetin on SBP in SHR

**Figure 6:**
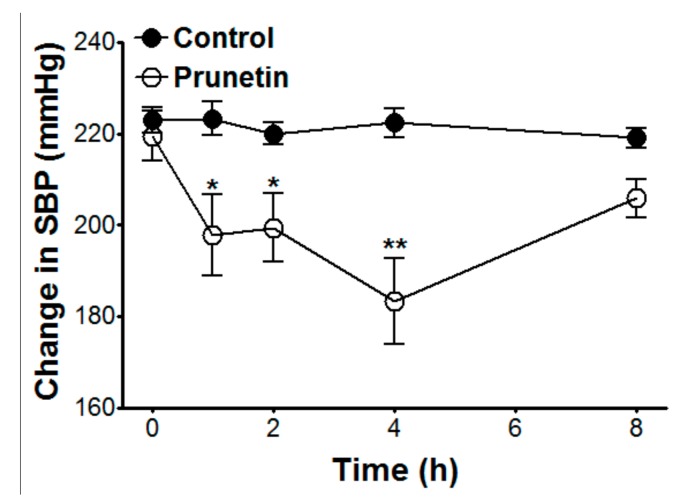
Hypotensive effect of prunetin on the changes in systolic blood pressure (SBP) of SHR. SBP was measured by using the non-invasive tail cuff system. The values are expressed as the mean ± SEM (*n* = 6). * *p* < 0.05, ** *p* < 0.01 vs. control.

Prunetin (25 mg/kg) significantly altered SBP by −21.6 ± 6.8, −20.0 ± 5.9, and −36.0 ± 10.6 mmHg at 1, 2, and 4 h after administration, respectively (Table 1, Figure 6).

## 3. Materials and Methods 

### 3.1. Chemicals and Reagents

Prunetin (≥98.0% purity by thin layer chromatography (TLC), CAS No: 552-59-0), modified KH buffer powder, PE, KCl, glibenclamide, 4-AP, TEA, calcium chloride (CaCl_2_), ethylene glycol-bis (2-aminoethylether)-*N*,*N*,*N*′,*N*′-tetra acetic acid (EGTA), caffeine, 2-APB, Y-27632, and dimethyl sulfoxide (DMSO) were purchased from Sigma Aldrich, Inc. (St. Louis, MO, USA). All other reagents were of analytical grades.

### 3.2. Animals and Preparation of Rat Aortic Rings

Sprague–Dawley rats (male, 240–260 g, 8 weeks old) and spontaneously hypertensive rats (SHR, male, 200–250 g, 8 weeks old) were purchased from Raonbio (Yongin city, Gyeonggi province, Korea). The animals were housed under standard laboratory conditions (22 °C ± 2 °C; lighting, 07:00–19:00) and were given free access to pelleted food and water for at least 1 week prior to use in experiments. All procedures were executed in accordance with the relevant animal welfare guidelines and were approved by the Kyung Hee University Institutional Animal Care and Use Committee [KHUASP(SE)-15-117]. The detailed methodology of the isolation and preparation of rat thoracic aorta used in this study has been described previously [25].

### 3.3. Experimental Protocols

#### 3.3.1. Effects of Prunetin with or without the Vascular Endothelium

The aortic rings, with or without the endothelium, were pre-contracted by PE (1 μM) in a standard KH buffer. After an equilibration period of 40 min, cumulative concentrations of prunetin (1, 3, 10, and 30 μg/mL) were investigated. Prunetin induced relaxation in the aortic rings, which was calculated as a percentage of the relaxation in response to PE.

#### 3.3.2. Effects of Prunetin on Extracellular Ca^2+^-Induced Contraction

We investigated the vasorelaxant effect of prunetin (1, 3, and 10 μg/mL) on extracellular Ca^2+^-induced contractions via receptor-operative Ca^2+^ channels (ROCCs) or voltage-dependent Ca^2+^ channels (VDCCs). The vascular contraction response was induced by extracellular CaCl_2_ (0.3–10 mM) in endothelium-denuded aortic rings that were pre-treated by PE (1 μM) or KCl (60 mM) in a Ca^2+^-free KH buffer with or without prunetin pre-incubation for 10 min. ROCCs and VDCCs can be activated by PE and KCl, respectively. The results were calculated as percentages; the control was the sample in the absence of prunetin pre-treatment.

#### 3.3.3. Effects of Prunetin on Intracellular Ca^2+^ Release

We investigated the inhibitory effect of prunetin on intracellular Ca^2+^ release using 2-APB and caffeine. After pre-incubation for 20 min with 2-APB (10 μM) before the induction of contractions by PE (1 μM), the effects of cumulative concentrations of prunetin (1–30 μg/mL) were investigated on endothelium-intact aortic rings in a normal KH solution. In another experiment, 20 min after the addition of prunetin (10 μg/mL), the contractile effects of caffeine (10 mM), a classic RyR agonist, were examined on endothelium-denuded aortic rings in a Ca^2+^-free KH containing EGTA (1 mM).

#### 3.3.4. Effects of Prunetin on the Rho-kinase Pathway 

We investigated the effect of prunetin on the Rho-kinase pathway. Fifteen minutes after the Rho-kinase inhibitor Y-27632 (1 μM), endothelium-denuded aortic rings were contracted by PE (1 μM), and then prunetin (1–30 μg/mL) was cumulatively added to the baths. The results were calculated as percentages and the control was the sample pre-injected with Y-27632.

#### 3.3.5. Effects of Prunetin on Aortic Rings with Vascular Endothelium Pre-Treated by Various K^+^ Channel Blockers

The effect of prunetin (1–30 μg/mL) on endothelium-intact aortic rings was investigated using a K^+^ channel blocker such as glibenclamide (10 μM), 4-AP (1 mM), or TEA (5 mM) for 20 min, before PE (1 μM) pre-contraction. The vasorelaxant effect of prunetin was calculated as a percentage of the relaxation in response to pre-treatment with K^+^ channel blockers.

#### 3.3.6. Effects of Prunetin on Blood Pressure in SHR

The SBP of SHR was measured by using a tail cuff method (CODA 8-Channel High Throughput Non-Invasive Blood Pressure System, Kent Scientific Co. Ltd., Torrington, CT, USA). SHR were allowed to acclimatize inside the restraint for 15 min before a blood pressure measurement was started. The changes in blood pressure were calculated by the subtraction of the blood pressure prior to administration from the blood pressure recorded at each hourly measurement. The SBP of SHR was measured before administration and 1, 2, 4, and 8 h after drug administration. The standard drug used was prunetin (25 mg/kg, orally). Prunetin dissolved in distilled water was used in this experiment. The control group was administered only distilled water. Six SHR received prunetin and six SHR received distilled water as controls through oral administration.

### 3.4. Statistical Analysis

All data are expressed as mean ± standard error of mean (SEM) and analyzed using IBM SPSS statistical analysis software, version 23.0 (SPSS Inc., Chicago, IL, USA). Comparisons of two groups were performed using Student’s *t*-test. Results with *p* values less than 0.05 were considered statistically significant.

## 4. Discussion

In the present study, prunetin relaxed both PE-contracted endothelium-intact and endothelium-denuded rat aortic rings; there was no difference in the vasorelaxant effect. The blood vessel contraction and relaxation were due to the action of the vascular endothelium and vascular smooth muscles. The vascular endothelium continuously releases a number of vasoactive hormones in response to blood flow, namely, vasoconstriction factors, such as endothelin, superoxide (O_2_^–^), and thromboxane, as well as vasodilation factors, such as nitric oxide (NO), prostaglandins, and endothelium-derived hyperpolarization factors (EDHF) [26,27]. Our findings suggested that the vasorelaxant effects of prunetin were not associated with any of these vascular endothelial-related mechanisms.

In the vascular smooth muscles, calcium channels are the most important and effective factors for the relaxation of blood vessels. Therefore, calcium channel blocker (CCB) drugs are the most frequently prescribed of the antihypertensive drug classes in the hospital [28]. The degree of muscle contraction is promoted by the cytosolic Ca^2+^ concentration, which induces the activation of the myofilament proteins, actin and myosin. The control of vascular smooth muscle contraction was regulated by different signal transduction pathways to increase the intracellular calcium concentration, namely the changes in the intracellular calcium concentration induced by intracellular Ca^2+^ release from the sarcoplasmic reticulum (SR) into cytoplasm by the IP_3_R and RyR channels, and extracellular Ca^2+^ influx via receptor operated calcium channels (ROCCs) or voltage dependent calcium channels (VDCCs) [29]. 2-APB inhibits Ca^2+^ entry through IP_3_R channels [30], store-operated Ca^2+^ channel (SOCC) [31], and SR Ca^2+^–ATPase pump [32], and caffeine activates all RyR channels to cause Ca^2+^ release from the SR [33]. In this study, pre-incubation with prunetin (3 and 10 μg/mL) inhibited vasoconstriction induced by Ca^2+^ of rat aortic rings pre-contracted with PE or KCl in a Ca^2+^-free KH buffer. These results suggested that prunetin inhibited the influx of extracellular Ca^2+^ via ROCCs or VDCCs. The cumulative concentrations of prunetin (1–30 μg/mL) exhibited a vasorelaxant effect, irrespective of 2-APB (10 μM) pre-incubation in KH buffer, but prunetin (10 μg/mL) pre-treatment inhibited caffeine-induced (10 mM) contraction of aortic rings in a Ca^2+^-free KH buffer. These results suggested that prunetin could inhibit Ca^2+^-induced vasoconstriction through the RyR channels, but not the IP_3_R channels, SOCC and SR Ca^2+^–ATPase pump. In the present study, we found that prunetin could inhibit vasocontraction by blocking ROCCs, VDCCs, and RyR channels. In addition, smooth muscle activity is regulated by both the cytosolic Ca^2+^ concentration through myosin light-chain kinase and the Ca^2+^ sensitivity of the myofilaments through myosin phosphatase [34]. The latter mechanism was related to the small GTPase Rho and one of its targets, Rho-kinase. As a pyridine derivative, Y-27632 selectively blocked the Ca^2+^ sensitization through the inhibition of Rho kinase [35]. In this study, pre-treatment of Y-27632 did not significantly affect the relaxant effects of prunetin. This result suggested that the vasorelaxant effect of prunetin was not associated with the Rho-kinase pathway.

In the present study, the vasorelaxant effects of prunetin were not altered by pre-treatment with 4-AP, TEA, and glibenclamide. The human genome contains more than 75 different potassium channel genes, and the potassium channels are the largest and most complex family of ion channels. Four major types of potassium channels are expressed in vascular smooth muscle cells: adenosine triphosphate (ATP)-sensitive (K_ATP_) channel, voltage gated (K_V_) channel, large conductance Ca^2+^-activated (BK_Ca_) channel, and inwardly rectifying (K_IR_) channel. 4-AP, TEA, and glibenclamide are well-known K_V_ channel, BK_Ca_ channel, and K_ATP_ channel blockers, respectively [36]. Our findings suggested that the vasorelaxant effects of prunetin were not related to the opening of these K^+^ channels.

To evaluate the blood pressure lowering effect of prunetin, the SBP of SHR was measured by using a cuff assay. At 1, 2, and 4 h after the oral administration of prunetin (25 mg/kg), the SBP of SHR was significantly decreased. These results were indicative of the antihypertensive effect of prunetin.

## 5. Conclusions

Prunetin is one representative of the isoflavone group in many foods and herbal medicines and exhibits many useful pharmacological effects. In the present study, we found that prunetin could lower blood pressure of SHR and relax isolated rat aortic rings through calcium channel block mechanisms in vessel smooth muscles. Therefore, in this study, prunetin has the potential for development as a functional ingredient or drug that could help to prevent or treat hypertension. However, in this study, the changes in the blood pressures (BP) of the SHR were measured only for 8 h by using a tail-cuff experiment to assess the antihypertensive effect of prunetin. To develop prunetin as a functional ingredient or drug for hypertension, further in vivo studies that include long-term (weeks) experiments on BP, direct BP measurement via installed catheters, and measurement of water electrolyte balance are needed.

## Figures and Tables

**Table 1:** Effects of prunetin on systolic blood pressure (SBP) in spontaneously hypertensive rats (SHR).

Groups	Control					Prunetin (25 mg/kg)				
Time (hour)	0	1	2	4	8	0	1	2	4	8
SBP (mmHg)	223.0 ± 2.8	223.3 ± 3.7	220.0 ± 2.5	222.3 ± 3.3	219.0 ± 2.2	219.5 ± 5.4	197.8 ± 8.9 *	199.4 ± 7.4 *	183 ± 9.4 **	205.8 ± 4.2

Values are expressed as mean ± SEM (*n* = 6). * *p* < 0.05, ** *p* < 0.01 vs. control.
